# Mid-arm muscle circumference as a significant predictor of all-cause mortality in male individuals

**DOI:** 10.1371/journal.pone.0171707

**Published:** 2017-02-14

**Authors:** Li-Wei Wu, Yuan-Yung Lin, Tung-Wei Kao, Chien-Ming Lin, Fang-Yih Liaw, Chung-Ching Wang, Tao-Chun Peng, Wei-Liang Chen

**Affiliations:** 1 Division of Family Medicine, Department of Family and Community Medicine, Tri-Service General Hospital; 2 School of Medicine, National Defense Medical Center, Taipei, Taiwan, Republic of China; 3 Division of Geriatric Medicine, Department of Family and Community Medicine, Tri-Service General Hospital; 4 Graduate Institute of Medical Sciences, National Defense Medical Center, Taipei, Taiwan, Republic of China; 5 Department of Otolaryngology–Head and Neck Surgery, Tri-Service General Hospital; and School of Medicine, National Defense Medical Center, Taipei, Taiwan, Republic of China; 6 Department of Pediatrics, Tri-Service General Hospital; and School of Medicine, National Defense Medical Center, Taipei, Taiwan, Republic of China; Leibniz-Institut fur Pflanzengenetik und Kulturpflanzenforschung Gatersleben, GERMANY

## Abstract

**Background:**

Emerging evidences indicate that mid-arm muscle circumference (MAMC) is one of the anthropometric indicators that reflect health and nutritional status, but its correlative effectiveness in all-cause mortality prediction of United States individuals remains uncertain.

**Methods and findings design:**

We investigated the joint association between MAMC and all-cause mortality in the US general population. A population-based longitudinal study of 6,769 participants aged 40 to 90 years in the third National Health and Nutrition Examination Survey (NHANES III) conducted by the National Center for Health Statistics of the Centers for Disease Control and Prevention. All participants were divided into two groups based on the gender: male and female group; each group was then divided into three subgroups depending on their MAMC level. The tertiles were as follows: T1 (18<27.3), T2 (27.3<29.6), T3 (29.6≤40.0) cm in the male group and T1 (15<22.3), T2 (22.3<24.6), T3 (24.6≤44.0) cm in the female group. Multivariable Cox regression analyses and Kaplan–Meier survival probabilities were utilized to jointly relate all-cause mortality risk to different MAMC level. For all-cause mortality in male participants, multivariable adjusted hazard ratios (HRs) were 0.83 (95% confidence interval (CI): 0.69–0.98; p = 0.033) for MAMC of 27.3–29.6 cm compared with 18–27.3 cm, and 0.76 (95% CI: 0.61–0.95; p = 0.018) for MAMC of 29.6–40 cm compared with 18–27.3 cm. For all-cause mortality in female participants, multivariable adjusted hazard ratios (HRs) were 0.84 (95% confidence interval (CI): 0.69–1.02; p = 0.075) for MAMC of 22.3–24.6 cm compared with 15–22.3 cm, and 0.94 (95% CI: 0.75–1.17; p = 0.583) for MAMC of 24.6–44 cm compared with 15–22.3 cm.

**Conclusion:**

Results support a lower MAMC is associated with a higher mortality risk in male individuals.

## Introduction

In general, age-related body composition changes lead to an increase in visceral fat and a decrease in muscle mass and fat-free mass [1–2]. Sarcopenia, defined as a loss of muscle strength and mass, is correlated with slow gait speed, poor physical performance, decreased mobility, and increased mortality [2]. The prevalence of sarcopenia and sarcopenic obesity in adults in the United States (US) increases with age and is highly prevalent according to data obtained from the National Health and Nutrition Examination Survey (NHANES), 1999–2004 [3]. It has been well documented that individuals with a lower fat mass and higher muscle mass have better survival outcomes, quality of life and mental health [1–4].

Recently, several anthropometric parameters and measurements, including waist circumference, hip circumference, triceps skinfold (TSF) thickness, mid-arm circumference (MAC), and mid-arm muscle circumference (MAMC), have been widely used for nutritional identification and risk prediction. MAMC, which has been demonstrated to be highly related to lean muscle mass measured by dual energy X-ray absorptiometry, is a simple and dependable anthropometric measurement and has excellent reliability when performed by well-trained personnel [4]. Several studies have also demonstrated that a lower MAMC is associated with a higher mortality risk, poorer mental health and quality of life, and poorer functional performance [2–6]. However, the association between MAMC and all-cause mortality in the US general population has not been widely investigated. This prompted us to use NHANES III data to investigate whether MAMC is a risk factor for all-cause mortality among middle-aged and older adults in the US.

## Materials and methods

### Study population

We selected adults between 40 and 90 years of age in the NHANES III, which represents a multistage stratified investigation of the US population living in households during 1988–1994 [7]. Demographic information was collected through a structured home interview and accompanied by a series of physical examinations, nutrition assessments, which included anthropometric measurements and body-composition assessments, and blood sampling at a mobile examination center (MEC). The NHANES III study was executed in accordance with the Declaration of Helsinki and approved by the National Center for Health Statistics (NCHS) Institutional Review Board after obtaining the written informed consent of participants before starting the study.

### Definition of the MAMC tertiles group

MAMC, as a marker of lean muscle mass, is calculated using the standard formula: MAMC = MAC–(3.14 x TSF thickness). The MAC was taken following standard procedures described by Lohman and colleagues and depending on the age of the participant [8]. Tertile-based analysis was used by dividing the MAMC into tertiles, with the participants in the lowest tertile as the reference group. All participants were initially divided into two groups based on gender, and then each group was divided into three subgroups (highest, middle and lowest, below and above cut-off level, respectively) depending on their MAMC level. The tertiles were as follows: T1 (18<27.3), T2 (27.3<29.6), T3 (29.6≤40.0) cm in the male group and T1 (15<22.3), T2 (22.3<24.6), T3 (24.6≤44.0) cm in the female group.

### Follow-up data on all-cause mortality

The NHANES III also contained detailed mortality information and follow-up data from the time of study participation. The follow-up data on all-cause mortality were provided by the NCHS according to the probabilistic matching between National Death Index death certificate records and NHANES III participants. The complete follow-up data for all participants on all-cause mortality in the NHANES III study were from the date of exam until the date of death or censoring on December 31, 2006 [9].

### Covariates

The participants were interviewed to collect information concerning age, gender, race/ethnicity, body measurements, blood pressure, medical conditions (including self-reported physician-diagnosed hypertension, diabetes mellitus, malignancy, stroke, congestive heart failure, cognitive impairment, number of prescription medications taken), marital status and smoking status. After collecting three or four blood pressure (BP) measurements in the MEC and using a mercury sphygmomanometer during the home examinations, the average systolic and diastolic blood pressure readings were obtained. The presence of hypertension was defined by self-report of a physician’s diagnosis or an average BP ≧140/90 mmHg. Diabetes mellitus was defined by a self-report of a physician’s diagnosis or the presence of a random serum glucose level ≧200 mg/dL. Serum uric acid levels were measured via a Hitachi 737 automated multichannel chemistry analyzer (Boehringer Mannheim Diagnostics, Indianapolis, IN, USA). The serum biochemistry profile was measured by the Lipoprotein Analytical Laboratory at Johns Hopkins University, Baltimore, Maryland. Cognitive function was assessed from the result of simple reaction time test (SRTT: visual motor speed). Considering that treatments may affect the serum biochemistry and quantitative biomarkers, such as fasting glucose and lipid related measures, the hypertensive participants taking antihypertensive medications (N = 4103), hyperlipidemia treatments (N = 793), or diabetes mellitus treatments (N = 730) were excluded from the study. All measurements were completed with standardized methods and documented accuracy with respect to the Centers for Disease Control and Prevention (CDC) reference methods [9].

### Data analysis

All statistical procedures and analyses were implemented using SPSS version 18 (SPSS, Inc., Chicago, IL). The analytic data were executed using the Complex Samples procedure to adjust for the clusters and strata of the complex sample design, incorporating sampling weights and preventing incorrect estimates of variance. Quantitative parameters were indicated as the values of the mean and standard deviation (SD). Demographic characteristics were compared using the Independent t-test or Wilcoxon Rank sum test for continuous variables and the Chi-square test for discrete variables. Two-sided p values of less than 0.05 were considered significant. A survival analysis was performed to examine the association of the MAMC with all-cause mortality. Kaplan-Meier survival curves were plotted to ascertain the relationship between MAMC in participants and subsequent mortality. Associations between MAMC tertiles and end points were evaluated in multivariable Cox proportional hazard models. The Cox proportional hazard assumption was met for the Cox model in the present study. Covariates adjustment was performed by an extended-model approach: Model 1 was not adjusted for other variables; Model 2 was further adjusted for age, race, sex, BMI and waist circumference; Model 3 = Model 2+ serum total cholesterol, serum HDL, serum glucose, C-reactive protein, serum uric acid, and serum total bilirubin; Model 4 = Model 3 + systolic blood pressure, smoking, type 2 diabetes mellitus and congestive heart failure; Model 5 = Model 4 + serum albumin, marital status, and number of prescription medications taken.

## Results

The study population in the NHANES III database consisted of the 6,769 adults who completed the MAMC measurement and laboratory examinations. Of the participants, 3,373 (49.8%) were male and 3,396 (50.2%) were female. The mean MAMC in the male group was 28.5±2.8 cm, and the mean age was 60.4±13.4 years. The mean MAMC in the female group was 23.8±3.0 cm, and the mean age was 60.5±13.8 years. The clinical characteristics of the male and female study population by MAMC tertiles are summarized in Table 1 and Table 2. Male participants with higher MAMC tertiles tended to have a higher BMI and a larger waist circumference, higher diastolic blood pressure, higher serum total cholesterol level, lower serum HDL level, higher serum glucose level, lower C-reactive protein level, higher serum uric acid level and higher serum AST level than males in the lower MAMC tertile. Male participants in the higher MAMC tertile were less likely to be non-Hispanic white and to have malignancy or stroke than men in the lower MAMC tertile. Female participants in the higher MAMC tertile tended to have a higher BMI and a larger waist circumference, higher systolic and diastolic blood pressure, higher serum total cholesterol level, lower serum HDL level, higher serum glucose level, higher C-reactive protein level, higher serum uric acid level, and lower serum albumin level than women in the lower MAMC tertile. Female participants in the higher MAMC tertile were less likely to be non-Hispanic white and more likely to have type 2 diabetes mellitus and congestive heart failure than women in the lower MAMC tertile.

**Table 1:** Characteristics of male individuals.

Characteristic	Tertiles of mid-arm muscle circumference (cm)				
	T1(18<27.3cm)	T2(27.3<29.6cm)	T3(29.6≤40.0cm)	total	*P* value
	*n* = 1,169	*n* = 1,125	*n* = 1,079	*n* = 3,373	
Continuous variables					
Mid-arm muscle circumference (cm), mean (SD)	25.6(1.6)	28.6(0.7)	31.7(1.6)	28.5(2.8)	<0.001
Age (years), mean (SD)	66.4(13.6)	59.4(12.8)	54.9(11.1)	60.4(13.4)	<0.001
BMI (kg/m^2^), mean (SD)	24.2(3.3)	26.9(3.2)	30.3(4.1)	27.0(4.3)	<0.001
Waist circumference (cm), mean (SD)	93.0(10.3)	98.5(9.8)	105.4(11.3)	98.8(11.6)	<0.001
Systolic blood pressure (mmHg), mean (SD)	136.4(23.5)	133.0(21.1)	132.5(18.4)	134.0(21.3)	<0.001
Diastolic blood pressure (mmHg), mean (SD)	74.4(12.8)	76.9(11.7)	79.6(11.4)	76.9(12.2)	<0.001
Serum total cholesterol (mg/dL), mean (SD)	209.8(43.1)	213.2(41.9)	215.6(41.4)	212.8(42.2)	0.005
HDL (mg/dL), mean (SD)	49.1(15.7)	45.6(13.7)	43.8(13.4)	46.2(14.5)	<0.001
Serum glucose, mean (SD)	105.1(36.2)	108.3(44.2)	109.0(41.7)	107.4(40.8)	0.046
C-reactive protein, mean (SD)	0.5(1.0)	0.5(0.8)	0.4(0.6)	0.5(0.8)	0.003
Serum uric acid, mean (SD)	5.8(1.4)	6.1(1.4)	6.4(1.3)	6.1(1.4)	<0.001
AST (U/L), mean (SD)	23.0(14.0)	23.3(15.7)	24.6(12.9)	23.6(14.3)	0.019
Serum total bilirubin, mean (SD)	0.7(0.3)	0.7(0.3)	0.7(0.3)	0.7(0.3)	0.463
Serum albumin (g/dL), mean (SD)	4.1(0.4)	4.2(0.3)	4.2(0.3)	4.2(0.4)	<0.001
Categorical variables					
Non-Hispanic white, *n* (%)	661(56.5)	575(51.1)	445(41.2)	1,681(49.8)	<0.001
Type 2 diabetes mellitus, *n* (%)	107(9.2)	117(10.4)	125(11.6)	349(10.3)	0.121
Malignancy, *n* (%)	72(6.2)	47(4.2)	28(2.6)	147(4.4)	0.001
Stroke, *n* (%)	68(5.8)	48(4.3)	26(2.4)	142(4.2)	0.001
Congestive heart failure, *n* (%)	75(6.4)	56(5.0)	44(4.1)	175(5.2)	0.117
Smoking, *n* (%)	330(28.2)	356(31.6)	316(29.3)	1002(29.7)	0.189
Depression, *n* (%)	57(4.9)	51(4.5)	47(4.4)	155(4.6)	0.884
Cognitive impairment, *N* (%)	42(26.9)	51(19.9)	60(17.2)	153(20.1)	0.042
Marital status, *N* (%)					<0.001
Married, *N* (%)	820(69.3)	845(75.1)	831(77.0)	2,486(73.7)	
Widowed, *N* (%)	22(1.9)	29(2.6)	17(1.6)	68(2.0)	
Divorced, *N* (%)	13(1.1)	28(2.5)	31(2.9)	72(2.1)	
Separated, *N* (%)	133(11.4)	60(5.3)	43(4.0)	236(7.0)	
Never married, *N* (%)	83(7.1)	89(7.9)	75(7.0)	247(7.3)	
Living with partner, *N* (%)	30(2.6)	23(2.0)	31(2.9)	84(2.5)	
Number of prescription medications taken, *N* (%)					0.153
<5	548(88.1)	491(90.6)	477(91.4)	1516(89.9)	
> = 5	74(11.9)	51(9.4)	45(8.6)	170(10.1)	

HDL, High-density lipoprotein; AST, aspartate aminotransferases; SD, standard deviation.

**Table 2:** Characteristics of female individuals.

Characteristic	Tertiles of mid-arm muscle circumference (cm)				
	T1(15<22.3cm)	T2(22.3<24.6cm)	T3(24.6≤44.0cm)	total	*P* value
	*n* = 1,111	*n* = 1,142	*n* = 1,143	*n* = 3,396	
Continuous variables					
Mid-arm muscle circumference (cm), mean (SD)	20.9(1.1)	23.4(0.7)	27.0(2.4)	23.8(3.0)	<0.001
Age (years), mean (SD)	61.0(14.7)	60.1(13.8)	60.3(12.8)	60.5(13.8)	0.245
BMI (kg/m^2^), mean (SD)	23.3(3.4)	27.1(3.7)	32.3(5.2)	27.6(5.6)	<0.001
Waist circumference (cm), mean (SD)	83.6(9.4)	92.9(9.8)	103.9(11.4)	93.6(13.2)	<0.001
Systolic blood pressure (mmHg), mean (SD)	130.4(25.0)	132.6(23.6)	136.8(24.1)	133.3(24.4)	<0.001
Diastolic blood pressure (mmHg), mean (SD)	69.3(14.3)	71.4(13.3)	73.6(12.8)	71.4(13.6)	<0.001
Serum total cholesterol (mg/dL), mean (SD)	220.9(44.0)	225.0(45.8)	225.4(46.1)	223.8(45.4)	0.036
HDL (mg/dL), mean (SD)	59.7(17.5)	55.3(15.3)	51.9(14.9)	55.6(16.3)	<0.001
Serum glucose, mean (SD)	100.0(40.9)	103.3(40.4)	116.2(53.5)	106.6(45.9)	<0.001
C-reactive protein, mean (SD)	0.4(0.8)	0.5(0.9)	0.7(0.8)	0.5(0.9)	<0.001
Serum uric acid, mean (SD)	4.6(1.3)	4.9(1.3)	5.3(1.5)	4.9(1.4)	<0.001
AST (U/L), mean (SD)	20.5(9.2)	21.3(13.4)	21.0(10.3)	20.9(11.1)	0.194
Serum total bilirubin, mean (SD)	0.5(0.2)	0.50(0.2)	0.5(0.2)	0.5(0.2)	0.247
Serum albumin (g/dL), mean (SD)	4.1(0.3)	4.1(0.3)	4.0(0.3)	4.0(0.3)	<0.001
Categorical variables					
Non-Hispanic white, *n* (%)	695(62.6)	608(53.2)	471(41.2)	1,774(52.2)	<0.001
Type 2 diabetes mellitus, *n* (%)	77(6.9)	103(9.0)	215(18.8)	395(11.6)	<0.001
Malignancy, *n* (%)	79(7.1)	65(5.7)	63(5.5)	207(6.1)	0.291
Stroke, *n* (%)	41(3.7)	34(3.0)	40(3.5)	115(3.4)	0.742
Congestive heart failure, *n* (%)	38(3.4)	33(2.9)	47(4.1)	118(3.5)	0.023
Smoking, *n* (%)	8(0.7)	4(0.4)	8(0.7)	20(0.6)	0.432
Depression, *n* (%)	98(8.8)	104(9.1)	87(7.6)	289(8.5)	0.553
Cognitive impairment, *N* (%)	66(27.0)	83(31.0)	105(40.9)	254(33.0)	0.003
Marital status, *N* (%)					0.018
Married, *N* (%)	579(52.1)	606(53.1)	559(48.9)	1,744(51.4)	
Widowed, *N* (%)	15(1.4)	19(1.7)	19(1.7)	53(1.6)	
Divorced, *N* (%)	14(1.3)	23(2.0)	23(2.0)	60(1.8)	
Separated, *N* (%)	291(26.2)	287(25.1)	288(25.2)	866(25.5)	
Never married, *N* (%)	109(9.8)	116(10.2)	125(10.9)	350(10.3)	
Living with partner, *N* (%)	30(2.7)	42(3.7)	58(5.1)	130(3.8)	
Number of prescription medications taken, *N* (%)					0.168
<5	599(89.1)	614(89.5)	652(86.6)	1,865(88.3)	
> = 5	73(10.9)	72(10.5)	101(13.4)	246(11.7)	

HDL, High-density lipoprotein; AST, aspartate aminotransferases; SD, standard deviation.

**Fig 1:**
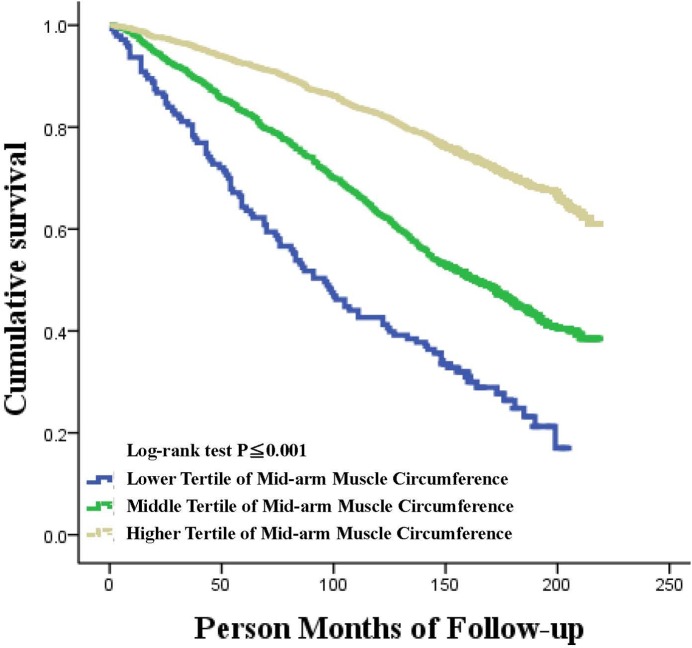
Kaplan Meier plot of association of mid-arm muscle circumference tertiles with all-cause mortality in the US male individuals.

**Fig 2:**
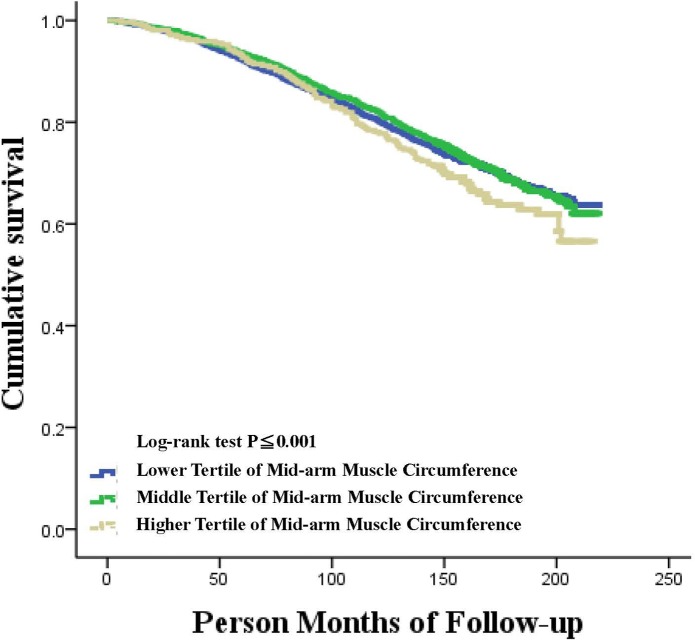
Kaplan Meier plot of association of mid-arm muscle circumference tertiles with all-cause mortality in the US female individuals.

In this study, the median length of follow-up was 14.3 years. A total of 2,493 deaths (1,396 male and 1,097 female) occurred during the follow-up. The unadjusted association of the MAMC tertiles with all-cause mortality in male and female participants are shown in Figs 1 and 2. The higher MAMC tertile had higher cumulative survival in male participants compared with the lower MAMC tertile, but this was not the case in female participants. Concerning the all-cause mortality in the male (shown in Table 3) and female (shown in Table 4) participants, a significant association was observed in male participants between the highest tertile of MAMC and all-cause mortality in both the univariable and multivariable adjusted analyses compared with the lowest tertile of MAMC, but this association was not observed in female participants.

**Table 3:** Cox proportional hazards regression of all-cause mortality for mid-arm muscle circumference in the US male individuals.

Models ^a^	Tertiles of mid-arm muscle circumference	Hazard Ratio(95%CI)	*P* -value
Model 1	• T2 v.s. T1 • T3 v.s. T1	• 0.53 (0.45–0.61) • 0.37 (0.31–0.44)	• <0.001 • <0.001
Model 2	• T2 v.s. T1 • T3 v.s. T1	• 0.82(0.69–0.97) • 0.76(0.61–0.94)	• 0.019 • 0.014
Model 3	• T2 v.s. T1 • T3 v.s. T1	• 0.82(0.69–0.97) • 0.77(0.61–0.96)	• 0.022 • 0.020
Model 4	• T2 v.s. T1 • T3 v.s. T1	• 0.80(0.67–0.95) • 0.75(0.60–0.94)	• 0.012 • 0.013
Model 5	• T2 v.s. T1 • T3 v.s. T1	• 0.83(0.69–0.98) • 0.76(0.61–0.95)	• 0.033 • 0.018

^a^ Adjusted covariates:

Model 1 = Unadjusted.

Model 2 = Adjustment for age, race, sex, BMI and waist circumference.

Model 3 = Model 2 + serum total cholesterol, serum HDL, serum glucose, C-reactive protein, serum uric acid, and serum total bilirubin.

Model 4 = Model 3 + systolic blood pressure, smoking, type 2 diabetes mellitus, congestive heart failure.

Model 5 = Model 4 + (serum albumin, marital status, number of prescription medications taken).

**Table 4:** Cox proportional hazards regression of all-cause mortality for mid-arm muscle circumference in the US female individuals.

Models ^a^	Tertiles of mid-arm muscle circumference	Hazard Ratio(95%CI)	*P* -value
Model 1	• T2 v.s. T1 • T3 v.s. T1	• 0.78 (0.65–0.94) • 0.88 (0.74–1.04)	• 0.008 • 0.125
Model 2	• T2 v.s. T1 • T3 v.s. T1	• 0.84(0.69–1.02) • 0.99(0.80–1.23)	• 0.074 • 0.940
Model 3	• T2 v.s. T1 • T3 v.s. T1	• 0.80(0.66–0.98) • 0.95(0.76–1.18)	• 0.027 • 0.632
Model 4	• T2 v.s. T1 • T3 v.s. T1	• 0.81(0.67–0.99) • 0.92(0.74–1.14)	• 0.036 • 0.450
Model 5	• T2 v.s. T1 • T3 v.s. T1	• 0.84(0.69–1.02) • 0.94(0.75–1.17)	• 0.075 • 0.583

^a^ Adjusted covariates:

Model 1 = Unadjusted.

Model 2 = Adjustment for age, race, sex, BMI and waist circumference.

Model 3 = Model 2 + serum total cholesterol, serum HDL, serum glucose, C-reactive protein, serum uric acid, and serum total bilirubin.

Model 4 = Model 3 + systolic blood pressure, smoking, type 2 diabetes mellitus, congestive heart failure.

Model 5 = Model 4 + (serum albumin, marital status, number of prescription medications taken).

## Discussion

In this study, we investigated a nationally representative sample of adults in the US population to determine whether there is an association between MAMC and all-cause mortality risk. The link between MAMC and all-cause mortality in US individuals has not been comprehensively evaluated. Most notably, this is the first study to demonstrate the relationship between MAMC and all-cause mortality in male and female individuals. These findings confirmed that a higher MAMC is associated with a lower risk of mortality in male individuals, with up to 24% lower risk for all-cause mortality, but there is no benefit in all-cause mortality in female individuals.

Emerging evidence has shown that MAMC is one of the main anthropometric parameters and distinguishing predictors of all-cause mortality in Asian and Western participants [2–6]. In a meta-analysis of prospective cohort studies of 35,287 participants, the overall mortality risk in participants with sarcopenic obesity defined by MAMC was increased by 46% (HR 1.46, 95% CI 1.23–1.73), compared with healthy participants [5]. Moreover, the results from the aging and longevity study in the Sirente geographic area [2] showed that in older adults, hazard ratios of mortality were 0.45 (95% confidence interval, 0.23–0.87), associated with the high tertile of MAMC. Previous research has also highlighted that high MAMC is a significant predictor of preserved physical performance and function, good quality of life and mental health [2,4]. These studies tend to indicate that MAMC is a significant predictor of all-cause mortality due to its applicability for routine use based on common clinical practice, and our results significantly confirm another potential prediction of all-cause mortality in US male participants.

Plausible explanations for this positive association between MAMC and all-cause mortality risk in individuals are multifactorial, including a decline in total muscle or fat-free mass, disturbance of glucose and lipid metabolism, and decreased plasma fatty acid utilization and oxidation. First, a decline in total muscle or fat-free mass, which has been related to total mortality, may be profoundly modulated by a number of physiological factors and may contribute to a low MAMC measurement [2–6,10]. Another previous study revealed that poorer muscle strength was associated with more difficulties in performing the physical activities of daily living [11]. Moreover, decreased physical activity predicted a decline in muscle strength and an increased risk of losing muscle mass [12–13]. The presence of an underlying medical illness (e.g., diabetes mellitus, strokes, and heart disease, including CAD) was also associated with decreased muscle mass and strength [14]. Proposed pathways of illness-related muscle impairment include physical inactivity, nutritional depletion, and systemic inflammation, which are risk factors associated with increased mortality rates [15–18]. Second, the disturbance of glucose and lipid metabolism has been found to play an important role in the association between MAMC and all-cause mortality risk. A decline in subcutaneous muscle might affect glucose and lipid metabolism, and another study has shown a relevance between intramuscular lipid and insulin resistance [19]. Additionally, previous research determined that higher glucose clearance and better preserved insulin sensitivity are detected in arm muscles than leg muscles, and these findings could result from differences in vascular responsiveness [20]. Third, reduced plasma fatty acid utilization and oxidation in the skeletal muscles of the arm have been reported in patients with obesity [21–23]. Patients with sarcopenic obesity, which is defined by both low muscle mass and obesity, might have an increased risk of mortality [5]. Recent studies extended the findings and demonstrated that impaired plasma free fatty acid utilization in forearm skeletal muscle and the activity of muscle oxidative enzymes were reduced in type 2 diabetes [21,23]. This may facilitate increased triacylglycerol storage and adipose tissue stores within skeletal muscle, leading to obesity, insulin resistance and hyperglycemia. Upper-body subcutaneous adipose tissue, measured by neck circumference, may be positively correlated with a high cardiovascular disease risk [24]. These research findings probably explain why the MAMC plays an important role in the prediction of all-cause mortality in the present study. These findings expand on our existing knowledge and strengthen the importance of using anthropometric assessment tools as potential clinical predictors of individuals at risk of all-cause mortality.

The plausible explanations for the presence of gender differences in muscle size-mortality relationships are biological differences in gender, including genetic factors, hormones effects and immune system responses, muscle capacity and physical function [25–28]. In general, men have higher total muscle mass and greater muscle capacity than women due to hormone effects, such as much higher levels of testosterone in men. Previous studies also demonstrated that males have greater muscle strength and higher physical performance, such as higher gait speed, than females [25–28]. Another explanation for MAMC predicting all-cause mortality in men but not women is women’s greater life expectancy, which would result in fewer deaths in women over the limited follow-up period. However, more evidence is still needed to explain the gender differences in muscle size-mortality relationships. In this study, we have explored evidence in support of the relationship between muscle size (MAMC) and all-cause mortality in male individuals. Early detection of decreased levels of MAMC may be beneficial in learning to recognize which male individuals are most in need of intervention to reduce their risk of mortality.

There were several potential limitations in this study. First, MAMC was collected at only one point during the follow-up period, which contributed to biased results. Second, self-reported medical conditions and the smoking status of individuals may on occasion have led to over-reporting and may be affected by recall bias or misclassification. Finally, despite adjustments having been made for a large number of potentially confounding factors, unmeasured confounders of the association between MAMC and the risk of all-cause mortality in US individuals cannot be ruled out.

In conclusion, the present study demonstrates that among male participants in a representative sample of the US population, a significant inverse association exists between MAMC and all-cause mortality risk. The easy, noninvasive measure of MAMC in community settings and hospitals suggests that it could be a simple, useful anthropometric tool for nutritional assessment and a clinical prognostic indicator for survival. Additional studies are warranted to further elucidate the mechanism(s) of this association.

## Supporting information

S1 File The minimal data set underlying the findings in our study. (XLS)

## References

[1] Woodrow G. Body composition analysis techniques in the aged adult: indications and limitations. Curr Opin Clin Nutr Metab Care. 2009; 12(1): 8–14. 10.1097/MCO.0b013e32831b9c5b 19057181

[2] Landi F, Russo A, Liperoti R, Pahor M, Tosato M, Capoluongo E, et al Midarm muscle circumference, physical performance and mortality: results from the aging and longevity study in the Sirente geographic area ilSIRENTE study. Clin Nutr. 2010; 29(4): 441–7. 10.1016/j.clnu.2009.12.006 20116909

[3] Batsis JA, Mackenzie TA, Jones JD, Lopez-Jimenez F, Bartels SJ. Sarcopenia, sarcopenic obesity and inflammation: Results from the 1999–2004 National Health and Nutrition Examination Survey. Clin Nutr. 2016. pii: S0261-5614(16)30020–6. 10.1016/j.clnu.2016.03.028 PMC6432912 27091774

[4] Noori N, Kopple JD, Kovesdy CP, Feroze U, Sim JJ, Murali SB, et al Mid-Arm muscle circumference and quality of life and survival in maintenance hemodialysis patients. Clin J Am Soc Nephrol. 2010; 5(12): 2258–68. 10.2215/CJN.02080310 20947789 PMC2994088

[5] Tian S, Xu Y. Association of sarcopenic obesity with the risk of all-cause mortality: A meta-analysis of prospective cohort studies. Geriatr Gerontol Int. 2016; 16(2): 155–66. 10.1111/ggi.12579 26271226

[6] Miller MD, Crotty M, Giles LC, Bannerman E, Whitehead C, Cobiac L, et al Corrected arm muscle area: an independent predictor of long-term mortality in community-dwelling older adults? J Am Geriatr Soc. 2002; 50(7): 1272–7. 12133024 10.1046/j.1532-5415.2002.50316.x

[7] Hyattsville, National Center for Health Statistics. Plan and operation of the Third National Health and Nutrition Examination Survey, 1988–94. Series 1: programs and collection procedures. Vital Health Stat 1. 1994; (32): 1–407. 7975354

[8] Lohman TG, Roche AF, Martorell R, eds. Anthropometric Standardization Reference Manual. Abridged ed. Champaign, IL: Human Kinetics Books; 1988.

[9] Hyattsville. The Third National Health and Nutrition Examination Survey (NHANES III) Linked Mortality File, Mortality follow-up through 2006: Matching Methodology.2009. Available at: http://www.cdc.gov/nchs/data/datalinkage/matching_methodology_nhanes3_final.pdf. (Accessed: 21 May 2014)

[10] Visser M, Goodpaster BH, Kritchevsky SB, Newman AB, Nevitt M, Rubin SM, et al Muscle mass, muscle strength, and muscle fat infiltration as predictors of incident mobility limitations in well-functioning older persons. J Gerontol A Biol Sci Med Sci. 2005; 60(3): 324–33. 15860469 10.1093/gerona/60.3.324

[11] Rantanen T, Guralnik JM, Sakari-Rantala R, Leveille S, Simonsick EM, Ling S, et al Disability, physical activity, and muscle strength in older women: The Women’s Health and Aging Study. Arch Phys Med Rehabil. 1999;80(2):130–5. 10025485 10.1016/s0003-9993(99)90109-0

[12] Rantanen T, Era P, Heikkinen E. Physical activity and the changes in maximal isometric strength in men and women from the age of 75 to 80 years. J Am Geriatr Soc. 1997;45(12):1439–45. 9400552 10.1111/j.1532-5415.1997.tb03193.x

[13] Roubenoff R. Sarcopenia and its implications for the elderly. Eur J Clin Nutrition. 2000;54 Suppl 3:S40–7. 11041074 10.1038/sj.ejcn.1601024

[14] Rantanen T, Masaki K, Foley D, Izmirlian G, White L, Guralnik JM. Grip strength changes over 27 yr in Japanese-American men. J Appl Physiol. 1998;85(6):2047–53. 9843525 10.1152/jappl.1998.85.6.2047

[15] Gosker HR, Wouters EF, van der Vusse GJ, Schols AM. Skeletal muscle dysfunction in chronic obstructive pulmonary disease and chronic heart failure: underlying mechanisms and therapy perspectives. Am J Clin Nutr. 2000;71(5):1033–47. 10799364 10.1093/ajcn/71.5.1033

[16] Corti MC, Guralnik JM, Salive ME, Sorkin JD. Serum albumin level and physical disability as predictors of mortality in older persons. JAMA. 1994;272(13):1036–42. 8089886

[17] Harris TB, Ferrucci L, Tracy RP, Corti MC, Wacholder S, Ettinger WH Jr, et al Associations of elevated interleukin-6 and C-reactive protein levels with mortality in the elderly. Am J Med. 1999;106(5):506–12. 10335721 10.1016/s0002-9343(99)00066-2

[18] Hakim AA, Petrovitch H, Burchfiel CM, Ross GW, Rodriguez BL, White LR, et al Effects of walking on mortality among nonsmoking retired men. N Engl J Med. 1998;338(2):94–9. 10.1056/NEJM199801083380204 9420340

[19] Hegarty BD, Furler SM, Ye J, Cooney GJ, Kraegen EW. The role of intramuscular lipid in insulin resistance. Acta Physiol Scand. 2003; 178(4): 373–83. 10.1046/j.1365-201X.2003.01162.x 12864742

[20] Olsen DB, Sacchetti M, Dela F, Ploug T, Saltin B. Glucose clearance is higher in arm than leg muscle in type 2 diabetes. J Physiol. 2005; 565(Pt 2): 555–62. 10.1113/jphysiol.2004.081356 15774531 PMC1464541

[21] Blaak EE, Wagenmakers AJ, Glatz JF, Wolffenbuttel BH, Kemerink GJ, Langenberg CJ, et al Plasma FFA utilization and fatty acid-binding protein content are diminished in type 2 diabetic muscle. Am J Physiol Endocrinol Metab. 2000; 279(1): E146–54. 10893334 10.1152/ajpendo.2000.279.1.E146

[22] Blaak EE, Wagenmakers AJ. The fate of [U-(13)C]palmitate extracted by skeletal muscle in subjects with type 2 diabetes and control subjects. Diabetes. 2002; 51(3): 784–9. 11872680 10.2337/diabetes.51.3.784

[23] Blaak EE. Basic disturbances in skeletal muscle fatty acid metabolism in obesity and type 2 diabetes mellitus. Proc Nutr Soc. 2004; 63(2): 323–30. 10.1079/PNS2004361 15294050

[24] Preis SR, Pencina MJ, D'Agostino RB Sr, Meigs JB, Vasan RS, Fox CS. Neck circumference and the development of cardiovascular disease risk factors in the Framingham Heart Study. Diabetes Care. 2013; 36(1): e3 10.2337/dc12-0738 23264305 PMC3526209

[25] Zeng P, Wu S, Han Y, Liu J, Zhang Y, Zhang E, et al Differences in body composition and physical functions associated with sarcopenia in Chinese elderly: reference values and prevalence. AChen LKrch Gerontol Geriatr. 2015; 60(1): 118–23. 10.1016/j.archger.2014.08.010 25440136

[26] Müller MJ, Geisler C, Pourhassan M, Glüer CC, Bosy-Westphal A. Assessment and definition of lean body mass deficiency in the elderly. Eur J Clin Nutr. 2014; 68(11): 1220–7. 10.1038/ejcn.2014.169 25139559

[27] Chen LK, Liu LK, Woo J, Assantachai P, Auyeung TW, Bahyah KS, et al Sarcopenia in Asia: consensus report of the Asian Working Group for Sarcopenia. J Am Med Dir Assoc. 2014; 15(2): 95–101. 10.1016/j.jamda.2013.11.025 24461239

[28] Cruz-Jentoft AJ, Baeyens JP, Bauer JM, Boirie Y, Cederholm T, Landi F, et al Sarcopenia: European consensus on definition and diagnosis: Report of the European Working Group on Sarcopenia in Older People. Age Ageing. 2010; 39(4): 412–23. 10.1093/ageing/afq034 20392703 PMC2886201

